# FBXW7 suppresses HMGB1-mediated innate immune signaling to attenuate hepatic inflammation and insulin resistance in a mouse model of nonalcoholic fatty liver disease

**DOI:** 10.1186/s10020-019-0099-9

**Published:** 2019-06-18

**Authors:** Cheng Zhang, Feng Chen, Li Feng, Qun Shan, Gui-Hong Zheng, Yong-Jian Wang, Jun Lu, Shao-Hua Fan, Chun-Hui Sun, Dong-Mei Wu, Meng-Qiu Li, Bin Hu, Qing-Qing Wang, Zi-Feng Zhang, Yuan-Lin Zheng

**Affiliations:** 1 School of Life Science, Jiangsu Normal University, Xuzhou, 221116 Jiangsu Province People’s Republic of China; 2 Key Laboratory for Biotechnology on Medicinal Plants of Jiangsu Province, Jiangsu Normal University, Xuzhou, 221116 Jiangsu Province People’s Republic of China; 3 College of Health Science, Jiangsu Normal University, Xuzhou, 221116 Jiangsu Province People’s Republic of China

**Keywords:** FBXW7, HMGB1, Innate immunity, Metaflammation, Insulin resistance, NAFLD

## Abstract

**Background:**

Innate immune dysfunction contributes to the development and progression of nonalcoholic fatty liver disease (NAFLD), however, its pathogenesis is still incompletely understood. Identifying the key innate immune component responsible for the pathogenesis of NAFLD and clarifying the underlying mechanisms may provide therapeutic targets for NAFLD. Recently, F-box- and WD repeat domain-containing 7 (FBXW7) exhibits a regulatory role in hepatic glucose and lipid metabolism. This study aims to investigate whether FBXW7 controls high-mobility group box 1 protein (HMGB1)-mediated innate immune signaling to improve NAFLD and the mechanism underlying this action.

**Methods:**

Mice were fed a high-fat diet (HFD) for 12 or 20 weeks to establish NAFLD model. Hepatic overexpression or knockdown of FBXW7 was induced by tail-vein injection of recombinant adenovirus. Some Ad-FBXW7-injected mice fed a HFD were injected intraperitoneally with recombinant mouse HMGB1 to confirm the protective role of FBXW7 in NAFLD via inhibition of HMGB1.

**Results:**

FBXW7 improves NAFLD and related metabolic parameters without remarkable influence of body weight and food intake. Moreover, FBXW7 markedly ameliorated hepatic inflammation and insulin resistance in the HFD-fed mice. Furthermore, FBXW7 dramatically attenuated the expression and release of HMGB1 in the livers of HFD-fed mice, which is associated with inhibition of protein kinase R (PKR) signaling. Thereby, FBXW7 restrains Toll-like receptor 4 (TLR4) and receptor for advanced glycation end products (RAGE) signaling in HFD-fed mouse livers. In addition, exogenous HMGB1 treatment abolished FBXW7-mediated inhibition of hepatic inflammation and insulin resistance in HFD-fed mouse livers.

**Conclusions:**

Our results demonstrate a protective role of FBXW7 in NAFLD by abating HMGB1-mediated innate immune signaling to suppress inflammation and consequent insulin resistance, suggesting that FBXW7 is a potential target for therapeutic intervention in NAFLD development.

**Electronic supplementary material:**

The online version of this article (10.1186/s10020-019-0099-9) contains supplementary material, which is available to authorized users.

## Background

Substantial evidence from experimental and epidemiological studies highlights a close association between innate immune dysfunction and nonalcoholic fatty liver disease (NAFLD) (Cai et al. 2018; Luo et al. 2018; Nati et al. 2016). In addition to triggering a chronic low grade inflammation termed metaflammation, innate immune dysfunction contributes to the glucose and lipid metabolic disorders including insulin resistance and steatosis and other key pathogenic features of NAFLD, playing a crucial role in the development and progression of NAFLD (Cai et al. 2018; Luo et al. 2018; Nati et al. 2016). Thus, identifying the key innate immune component responsible for pathogenesis of NAFLD and clarifying the underlying mechanisms may provide therapeutic targets for NAFLD.

F-box- and WD repeat domain-containing 7 (FBXW7) is a substrate recognition subunit of the Skp1/Cull 1/F-box (SCF) ubiquitin ligase complex that mediates the ubiquitylation of diverse protein substrates, such as Notch, cyclin E, c-Myc, c-Jun and mammalian target of rapamycin (mTOR) for proteasomal degradation, controlling multiple physiological processes (Kanatsu-Shinohara et al. 2014; Kourtis et al. 2015; Zhao et al. 2016). It has been reported that the FBXW7 level is downregulated in the livers of obese mice and patients (Tu et al. 2012; Zhao et al. 2018). In addition, liver-specific Fbxw7 knockout mice exhibit an impaired glucose and lipid homeostasis due to the abolishment of FBXW7-mediated degradation of several protein substrates such as REV-ERBα and hepatokine Fetuin A (Onoyama et al. 2011; Zhao et al. 2016, 2018). These studies indicate a regulatory role of FBXW7 in metabolic diseases including NAFLD. Recently, FBXW7 has been implicated in regulating innate immune response upon carcinogenesis and infection, such as repressing CCAAT/enhancer binding protein delta (C/EBPδ)-mediated inflammatory response and promoting antiviral immune response (Balamurugan et al. 2013). However, whether FBXW7 controls innate immune signaling to modulate NAFLD development and progression has yet to be investigated.

Damage-associated molecular patterns (DAMPs) and their specific receptors (termed “pattern recognition receptors”) play a crucial role in innate immune activation-mediated sterile inflammation under various pathological conditions including NAFLD (De Lorenzo et al. 2018; Mihm 2018). High-mobility group box 1 protein (HMGB1), a non-histone nuclear protein, translocates from the nucleus to the cytoplasm and is consequently released into extracellular milieu in response to cell damage, death and stress, acts as an important DAMP (Lu et al. 2014; Lotze and Tracey 2005). HMGB1 promotes the liver injuries and diseases via various pathways, such as binding to specific receptor, eg, Toll-like receptor 4 (TLR4) and receptor for advanced glycation end products (RAGE) and consequently provoking inflammatory response (Li et al. 2011; Lundbäck et al. 2016). There is growing evidence that the blockade of extracellular HMGB1 exhibits encouraging therapeutic effects on inflammatory diseases including NAFLD (Afrin et al. 2017; Entezari et al. 2014; Zhang et al. 2013a; Zeng et al. 2015), indicating that HMGB1 is a promising biomarker and therapeutic target for these diseases.

In this study, we hypothesized that FBXW7 ameliorates the development and progression of NAFLD through abating HMGB1-mediated innate immune signaling to suppress inflammation and consequent insulin resistance. This study provides the experimental evidence for the protective role of FBXW7 against innate immune dysfunction-mediated NAFLD development, as well as the mechanism underlying this action, indicating that FBXW7 is a potential target for therapeutic intervention in NAFLD.

## Methods

### Mice

All experimental and euthanasia procedures performed in this study were approved by the Institutional Animal Care and Use Committee of Jiangsu Normal University and complied with the ethical guidelines for the care and use of laboratory animals of the Chinese Ministry of Science and Technology.

6-week-old male C57BL/6 mice were purchased from Hua-fu-kang Biological Technology Co. Ltd. (Beijing, China). Mice were maintained in a 12 h light-dark cycle, at constant temperature (23 ± 1 °C) and humidity (60%), allowed free access to rodent food and water. After acclimation for two weeks, mice were fed a normal diet (ND, 10% of energy as fat; D12450B; Research Diets, New Brunswick, NJ, USA) or a HFD (High-fat diet, 60% of energy as fat; D12492; Research Diets, New Brunswick, NJ, USA) for 12 or 20 weeks.

Mice were fasted overnight, anaesthetized and sacrificed at the end of the feeding period. The liver, epididymal fat, muscles and blood were immediately collected for experiments or stored at − 80 °C until analysis.

### Injection of recombinant adenovirus

Recombinant adenovirus expressing FBXW7 (pHBAd-MCMV-GFP- Fbxw7) or green fluorescent protein (GFP) (pHBAd-MCMV-GFP) vector was purchased from Hanbio Biotechnology Co., Ltd. (Shanghai, China). 1 × 10^9^ plaque forming units (PFU) of Ad-FBXW7 or Ad-GFP in a final volume of 200 μl of sterile 0.9% NaCl were injected into the tail vein of mice at the end of 18 weeks of dietary manipulation.

Recombinant adenovirus harboring FBXW7 shRNA or Scramble shRNA was purchased from Hanbio Biotechnology Co., Ltd. (Shanghai, China). The FBXW7 shRNA sequence is as follows: 5′-GAGACTTCATCTCCTTGCTTCCTAA-3′. 1 × 10^9^ PFU of Ad-FBXW7 shRNA or Ad-Scramble shRNA in a final volume of 200 μl of sterile 0.9% NaCl were injected into the tail vein of mice at the end of 10 weeks of HFD feeding.

Oral glucose tolerance tests (OGTT) and insulin tolerance tests (ITT) were performed 10 days after adenoviral injections. Mice were fasted overnight, anaesthetized and sacrificed 2 weeks after adenoviral injections (at the end of the feeding period).

### Recombinant HMGB1 treatment

Recombinant mouse HMGB1 (purity: > 95%, endotoxin level: < 1.0EU per 1 μg) was purchased from Cloud-Clone Corp. (Houston, TX, USA). Some Ad-FBXW7-injected mice fed a HFD were injected intraperitoneally (IP) every other day for 14 days with HMGB1 (20 μg per mouse each time) at the end of 18 weeks of dietary manipulation. The mice received the first injection of HMGB1 at 6 h after Ad-FBXW7 injection. An equal volume of PBS was given to the control mice.

OGTT and ITT were performed 10 days after the first injection of HMGB1. Mice were fasted overnight, anaesthetized and sacrificed 2 weeks after the first injection (at the end of the feeding period).

### OGTT and ITT

OGTT and ITT were performed in fasted mice (16 h or 6 h) with oral administration of glucose (2 g of glucose per kg of body weight) or intraperitoneal (i.p.) injection of insulin (0.75 units of insulin per kg of body weight), respectively. Blood glucose values were measured with an Ascensia Elite glucose meter (Bayer Corporation, Mishawaka, IN, USA) by tail venipuncture immediately before (0 min) and after (15, 30, 60, 90, and 120 min) oral administration of glucose or insulin injection.

### Biochemical analyses

The levels of blood glucose were determined with an Ascensia Elite glucose meter by tail venipuncture. The serum alanine aminotransferase (ALT) activities were spectrophotometrically measured with a diagnostic kit (Jiancheng Institute of Biotechnology, Nanjing, China) following the manufacturer’s instructions.

Hepatic lipids were extracted from approximately 200 mg frozen liver samples using chloroform:methanol (2:1 v/v) solution, as described by Folch and Lees (Folch et al. 1957) and resuspended in phosphate-buffered saline (PBS) containing 5% Triton X-100 (Amresco, Solon, OH, USA). The serum sample and hepatic lipid extraction solution were used to determinate triglyceride (TG) levels using the corresponding LabAssay kit (Wako Chemicals, Richmond, VA, USA) according to the manufacturer’s instructions.

### Enzyme-linked immunosorbent assay

Serum insulin and HMGB1 levels were determined with the enzyme-linked immunosorbent assay kits (mouse insulin ELISA kit: ALPCO Diagnostics, Windham, NH, USA; mouse HMGB1 ELISA kit: Cloud-Clone Corp., Houston, TX, USA) following the manufacturer’s instructions.

### Liver slice collection and histopathological analysis

The preparation of frozen liver sections and hematoxylin-eosin staining were performed according to the protocols described in our previous work (Zhang et al. 2013b; Zhang et al. 2014). The liver sections stained with hematoxylin-eosin (Sigma-Aldrich, St. Louis, MO, USA) were examined by an expert liver pathologist blinded to the treatment groups.

### Immunohistochemistry

After antigen retrieval and endogenous peroxidase activity blocking, the frozen liver sections were blocked with 3% normal goat serum in 0.1% Triton-X100 in PBS for 1.5 h, then were incubated overnight at 4 °C with primary antibodies (Rabbit anti-HMGB1 antibody, 1:400, Abcam, Cambridge, MA, USA). The liver sections were rinsed three times with PBS, then were incubated with horseradish peroxidase (HRP)-conjugated goat anti-rabbit IgG for 1 h at room temperature. Positive signals were developed with DAB kit (Zsbio Commerce Store, Beijing, China), followed by counterstaining with hematoxylin. Stained specimens were captured using a leica DM4000B microscope (Leica Microsystems, Germany).

### Immunofluorescence staining and confocal imaging

The immunofluorescence staining were performed as described previously (Zhang et al. 2013b, 2014). Double/triple staining was performed by simultaneously adding one or two primary antibodies. The frozen liver sections were incubated overnight at 4 °C with the following primary antibody: rat anti-F4/80 antibody (1:500), rabbit anti-TLR4 antibody (1:300) or rabbit anti-RAGE antibody (1:200). After a washing with PBS, the liver sections were incubated with Alexa Fluor 647-conjugated donkey anti-rat IgG H&L and Alexa Fluor 568-conjugated goat anti-rabbit IgG H&L. All the above primary and secondary antibodies were purchased from Abcam (Cambridge, MA, USA). Staining specificity was assessed by omitting primary antibodies. Sections were counterstained with ProLong® Gold containing 4, 6-diamidino-2-phenylindole (Invitrogen, Carlsbad, CA, USA) according to the manufacturer’s instructions. Stained specimens were captured using a Leica TCS SP8 confocal microscope equipped with a Leica DMI 8 inverted microscope (Leica Microsystems, Germany).

### Quantitative real time polymerase chain reaction

The quantitative real time polymerase chain reaction was performed as described in our previous work (Zhang et al. 2014). The primers used were: F4/80: Forward, 5′- CTTTGGCTATGGGCTTCCAGTC-3′, Reverse, 5′- GCAAGGAGGACAGAGTTTATCGTG-3′; Interleukin-1β (IL-1β): Forward, 5′-AAATACCTGTGGCCTTGGGC-3′, Reverse, 5′-CTTGGGATCCACACTCTCCAG-3′; Tumor necrosis factor α (TNF-α): Forward, 5′-TCTCATTCCTGCTTGTGG-3′, Reverse, 5′-ACTTGG TGGTTTGCTACG-3′; Monocyte chemoattractant protein-1 (MCP-1): Forward, 5′-AGGTCCC TGTCATGCTTCTG-3′, Reverse, 5′-GCTGCTGGTGATCCTCTTGT-3′; Glyceraldehyde-3-phosphate dehydrogenase (GAPDH): Forward, 5′-AGGTCGGT GTGA ACGGATTTG-3′, Reverse, 5′-TGTAGACCATGTAGTTGAGGTCA-3′. The relative levels of target mRNAs, were normalized to GAPDH mRNA, and were calculated by the comparative cycle threshold (Ct) method.

### Western blot analysis

The preparation of liver tissue homogenates and western blot analyses were performed as described in our previous work (Zhang et al. 2013b, 2014). The liver cytoplasmic and nuclear extracts were obtained using a nuclear/cytoplasmic isolation kit (Pierce Biotechnology, Inc., Rockford, IL, USA). The following primary antibodies were used: rabbit anti-FBXW7 antibody (Thermo Fisher Scientific Inc., Waltham, MA, USA); rabbit anti-Akt, rabbit anti-p-Akt (Ser473), rabbit anti-glycogen synthase kinase-3β (GSK-3β), rabbit anti-p-GSK-3β (Ser9), rabbit anti-nuclear factor kappa B (NF-κB) p65, rabbit anti-myeloid differentiation factor 88 (MyD88), rabbit anti-extracellular-signal-regulated kinase (ERK), rabbit anti-p-ERK (Thr202/Tyr204), rabbit anti-JNK and rabbit anti-p-JNK (Thr183/Tyr185) antibodies (Cell Signaling Technology, Beverly, MA, USA); rabbit anti-HMGB1, rabbit anti- protein kinase R (PKR), rabbit anti-p-PKR (Thr451), rabbit anti-myeloid differentiation factor 2 (MD2) and mouse anti-GAPDH antibody, rabbit anti-Lamin A/C antibodies (Abcam, Cambridge, UK); rabbit anti-caspase-1p20, rabbit anti-caspase-1, mouse anti-TLR4 and mouse anti-RAGE antibodies (Santa Cruz Biotechnology, Santa Cruz, CA, USA). After washing, proteins were detected using HRP-conjugated anti-rabbit or HRP-conjugated anti-mouse secondary antibodies (Cell Signaling Technology, Beverly, MA, USA). Immunoreactive proteins were visualized using 20 × LumiGLO® Reagent and 20 × Peroxide (Cell Signaling Technology, Beverly, MA, USA). The optical density (OD) values of the detected bands were measured with Scion Image analysis software (Scion Corp., Frederick, MD, USA). The OD values were normalized using appropriate internal controls (optical density detected protein/optical density internal control).

### Statistical analysis

All statistical analysis was performed using SPSS version 22. Data were expressed as means ± standard deviation (SD) and were analyzed with two-tailed student’s t-test (Comparison between two groups) or a one-way analysis of variance (ANOVA) followed by Tukey’s Honestly Significant Difference (HSD) post-hoc test (data analysis of more than two groups). Statistical significance was set at *p* < 0.05.

## Results

### FBXW7 improves NAFLD and related metabolic parameters in HFD-fed mice

**Fig. 1:**
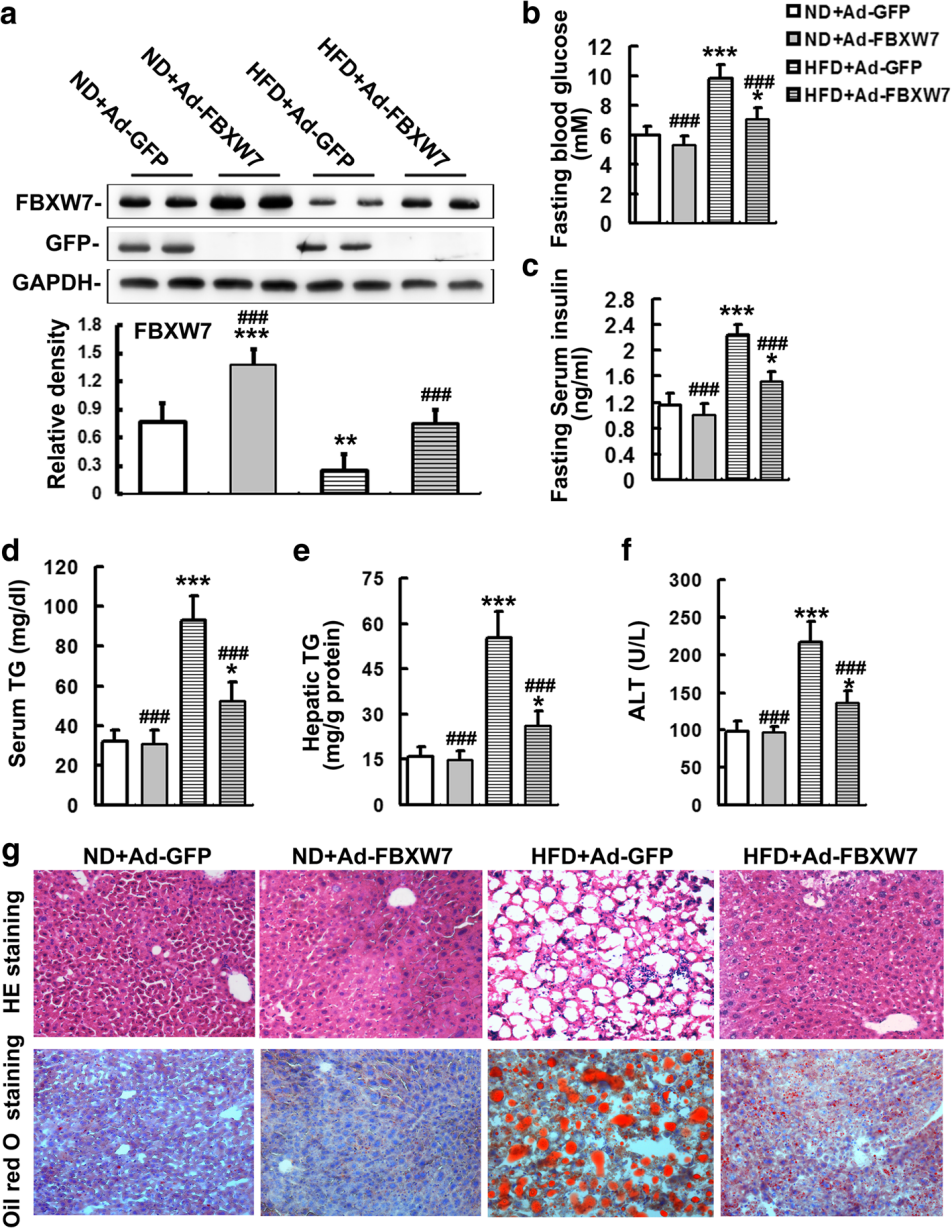
FBXW7 improves NAFLD and related metabolic parameters in HFD-fed mice (*n* = 5). **a** Immunoblotting and densitometry of FBXW7 and GFP in mouse livers. **b** The levels of fasting blood glucose in different treatment groups. **c** The levels of fasting serum insulin in different treatment groups. **d** Serum TG levels in different treatment groups. **e** Hepatic TG levels in different treatment groups. **f** Serum ALT activities in different treatment groups. **g** H&E staining and Oil Red O staining of liver sections in different treatment groups. Magnification 200X. All of the values are expressed as the mean ± SD. *P < 0.05, **P < 0.01, ***P < 0.001 versus the ND + Ad-GFP group; ###P < 0.001 versus the HFD + Ad-GFP group

Previous studies suggest that FBXW7 is down-regulated in NAFLD mouse models and patients (Tu et al. 2012; Zhao et al. 2018), indicating a regulatory role of FBXW7 in the development of NAFLD. To gain insights into the effects of FBXW7 on HFD-induced NAFLD and related metabolic features, GFP or FBXW7 expression adenovirus were injected intravenously into mice fed a ND or HFD. The expression of FBXW7 was remarkably augmented in the livers of FBXW7-injected mice relative to GFP-injected mice under both ND and HFD feeding conditions (Fig. 1a), but not in other metabolic tissues (i.e., skeletal muscles and white adipose tissues) (Additional file 1: Figure S1), confirming the effectiveness of FBXW7 overexpression in mouse livers. Our results showed that the protein levels of FBXW7 were dramatically diminished in the livers of GFP- injected mice fed a HFD compared with ND-fed controls (Fig. 1a), which is consistent with those reports (Tu et al. 2012; Zhao et al. 2018).

GFP- injected mice developed a significant obesity after 18 weeks HFD feeding (Additional file 2: Figure S2a). FBXW7 overexpression did not markedly affect body weight, as well as food intake, in both ND and HFD-fed mice (Additional file 2: Figure S2). There was a notable increase in the levels of fast blood glucose, serum insulin and TG in GFP-injected mice fed a HFD compared with ND-fed controls (Fig. 1b-d). FBXW7 overexpression remarkably decreased the levels of these metabolic parameters in HFD-fed mice (Fig. 1b-d). An evident liver injury including the elevation of serum ALT activity, hepatocyte hypertrophy and vacuolization, fat accumulation and inflammatory cell infiltration was apparent in the livers of GFP-injected mice fed a HFD, which was significantly ameliorated by FBXW7 overexpression (Fig. 1e-g). The results of Oil Red O staining and biochemical analysis also showed a marked steatosis occurred in the livers of GFP-injected mice fed a HFD, while it was notably attenuated in the livers of FBXW7-injected mice fed a HFD (Fig. 1e and g).

These results suggest that FBXW7 improves NAFLD and related metabolic parameters in HFD-fed mice.

### FBXW7 alleviates insulin resistance in HFD-fed mouse livers

**Fig. 2:**
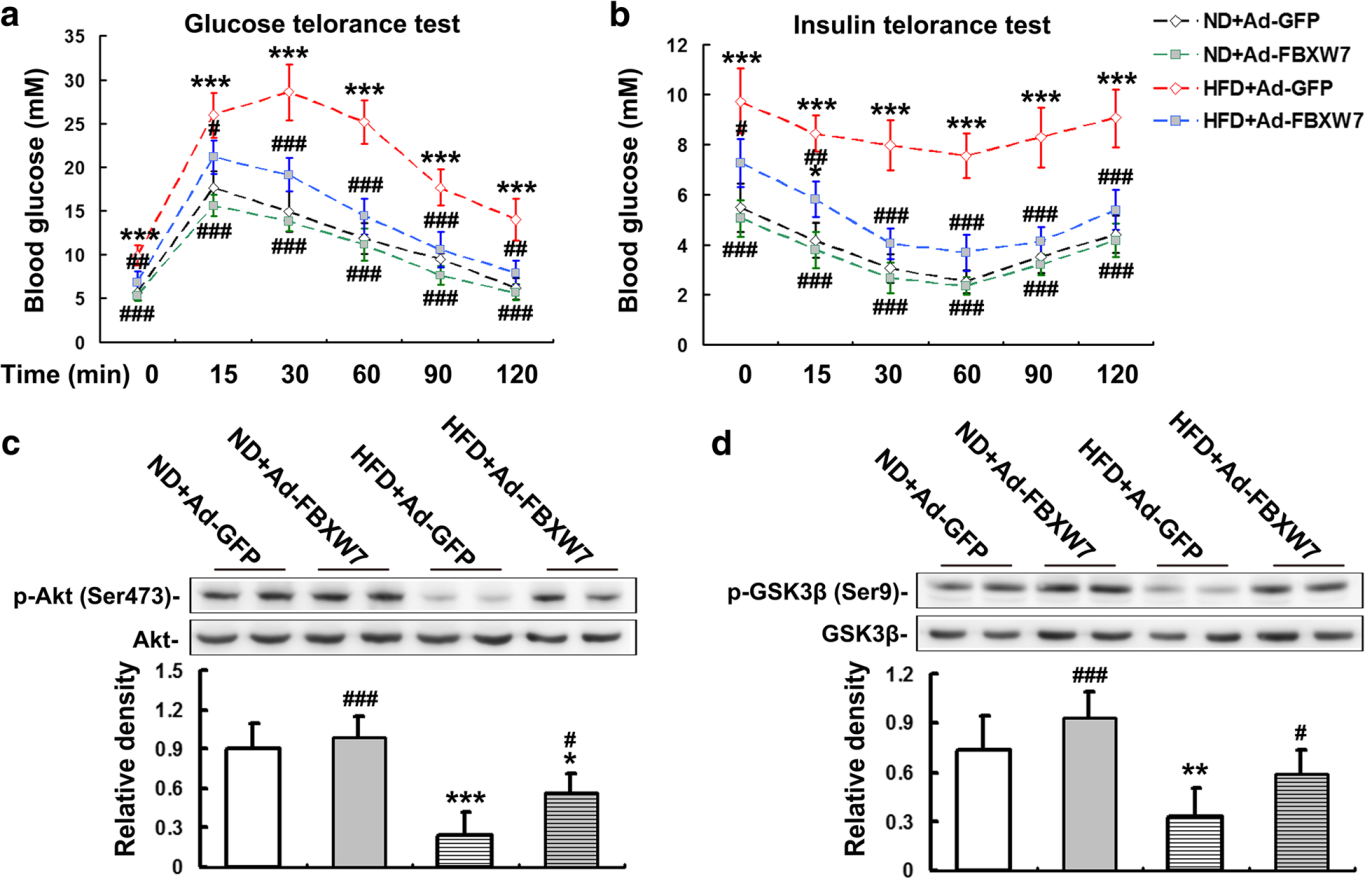
FBXW7 alleviates insulin resistance in HFD-fed mouse livers (*n* = 4). **a** Data of glucose tolerance tests in different treatment groups. **b** Data of insulin tolerance tests in different treatment groups. **c** Immunoblotting and densitometry of p-Akt in mouse livers. **d** Immunoblotting and densitometry of p-GSK3β in mouse livers. All of the values are expressed as the mean ± SD. *P < 0.05, **P < 0.01, ***P < 0.001 versus the ND + Ad-GFP group; #*P* < 0.05, ##*P* < 0.01, ###*P* < 0.001 versus the HFD + Ad-GFP group

It has been well established that insulin resistance is closely associated with NAFLD and plays an important role in the pathogenesis of latter (Gastaldelli 2017; Jelenik et al. 2017). We determined the hepatic insulin resistance status by OGTT, ITT and western blot analysis of hepatic insulin signaling components. The results of OGTT and ITT showed that GFP-injected mice fed a HFD exhibited a remarkable impairment of glucose tolerance and systemic insulin sensitivity, characterized by the markedly increased levels of blood glucose at all time points after glucose gavage or insulin injection, compared with GFP-injected mice fed a ND (Fig. 2a, b). However, FBXW7 overexpression effectively improved the glucose intolerance and systemic insulin resistance in HFD-fed mice (Fig. 2a, b). The results of western blot analysis revealed that HFD largely reduced the protein levels of p-Akt (Ser473) and p-GSK3β (Ser9) in the livers of GFP-injected mice, indicating an impairment of hepatic insulin signaling (Fig. 2c, d). Interestingly, the protein levels of p-Akt (Ser473) and p-GSK3β (Ser9) were largely augmented in the livers of FBXW7-injected mice fed a HFD compared to the GFP-injected mice fed a HFD (Fig. 2c, d).

Collectively, these results indicate that FBXW7 alleviates insulin resistance in HFD-fed mouse livers.

### FBXW7 attenuates inflammatory response in HFD-fed mouse livers

**Fig. 3:**
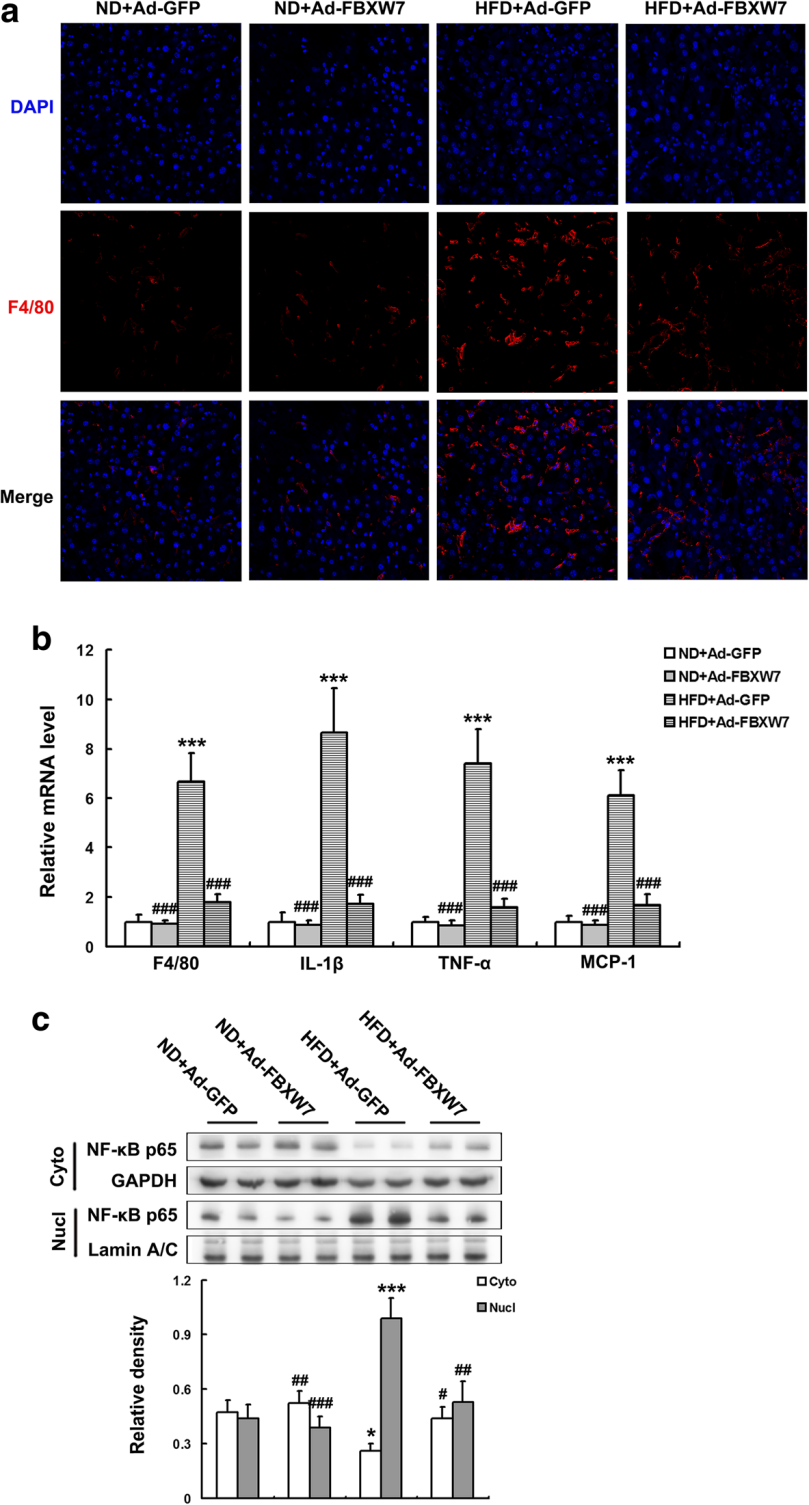
FBXW7 attenuates inflammatory response in HFD-fed mouse livers. **a** Representative confocal immunofluorescence images of F4/80 (red) and DAPI (blue) in mouse livers (*n* = 4). **b** The mRNA level of inflammation-related genes in mouse livers (*n* = 3). **c** Immunoblotting and densitometry of nuclear and cytoplasmic NF-κB p65 in mouse livers (*n* = 4). All of the values are expressed as the mean ± SD. *P < 0.05, ***P < 0.001 versus the ND + Ad-GFP group; #*P* < 0.05, ##*P* < 0.01, ###*P* < 0.001 versus the HFD + Ad-GFP group

Chronic inflammation leads to an impairment of insulin signaling, thereby contributing to the development of hepatic insulin resistance and the progression of NAFLD (Perry et al. 2015; Saberi et al. 2009; Tilg and Hotamisligil 2006). The results of immunofluorescence staining and Q-PCR showed that HFD promoted a dramatic inflammation as evident by the notable up-regulation of F4/80 (Kupffer cell marker) expression in the livers of GFP-injected mice fed a HFD compared to ND-fed controls (Fig. 3a, b). Whereas, FBXW7 overexpression markedly abated F4/80 levels in the livers of HFD-fed mice (Fig. 3a, b). Moreover, HFD notably elevated the expression of inflammation-related genes including IL-lβ, TNF-α and MCP-1 in the livers of GFP-injected mice, which was significantly suppressed by FBXW7 overexpression (Fig. 3b). NF-κB is the primary transcription factor responsible for the expression of inflammation-related genes under metaflammation conditions (Ke et al. 2015). Our results showed that the nuclear translocation of NF-κB p65, a marker of its transcriptional activation, was largely provoked by HFD feeding in the livers of GFP-injected mice compared to ND-fed controls (Fig. 3c). Interestingly, FBXW7 overexpression markedly decreased the nuclear translocation of NF-κB p65 in the livers of HFD-fed mice, indicating an inhibition of its transcriptional activation (Fig. 3c).

Taken together, these results indicate that FBXW7 attenuates inflammatory response in HFD-fed mouse livers.

### FBXW7 suppresses the expression and release of HMGB1 in HFD-fed mouse livers

**Fig. 4:**
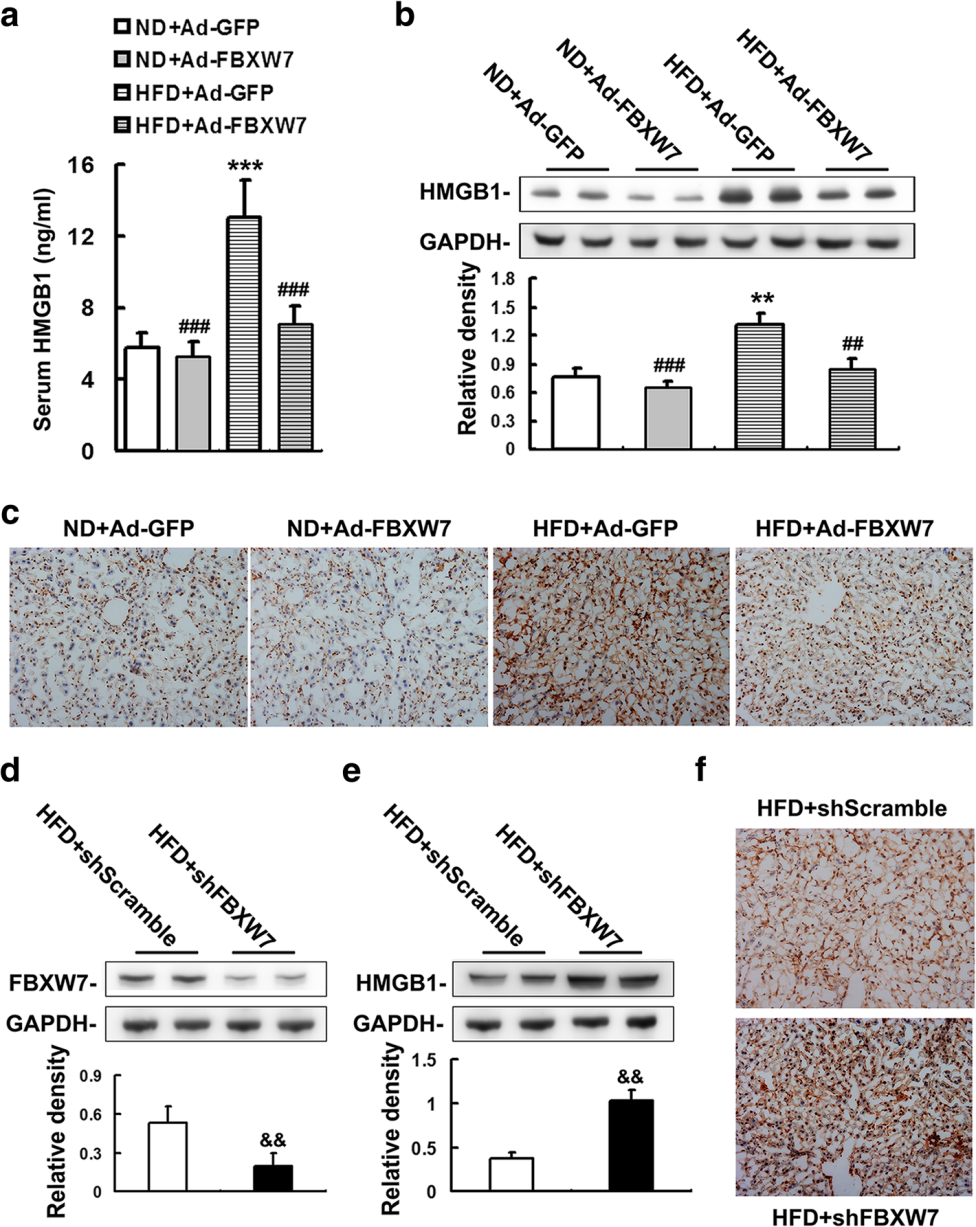
FBXW7 suppresses the expression and release of HMGB1 in HFD-fed mouse livers. **a** Serum HMGB1 levels in different treatment groups (*n* = 5). **b** and (**e**) Immunoblotting and densitometry of HMGB1 in mouse livers (*n* = 4). **c** and (**f**) Immunohistochemistry staining of HMGB1 in mouse livers (*n* = 4). **d** Immunoblotting and densitometry of FBXW7 in mouse livers (*n* = 4). Magnification 200X. All of the values are expressed as the mean ± SD. **P < 0.01, ***P < 0.001 versus the ND + Ad-GFP group; ##*P* < 0.01, ###*P* < 0.001 versus the HFD + Ad-GFP group; && *P* < 0.01 versus the HFD + shScramble group

Extracellular HMGB1, an endogenous danger molecule, has been reported to trigger innate immune activation-mediated inflammatory response under various pathological conditions, including NAFLD (Li et al. 2011; Lundbäck et al. 2016). A notably increased level of serum and hepatic HMGB1 was found in GFP-injected mice fed a HFD compared to ND-fed controls (Fig. 4a, b). Nevertheless, FBXW7 overexpression effectively lowered serum and hepatic HMGB1 levels in HFD-fed mice (Fig. 4a, b). To determine the cellular localization of hepatic HMGB1, we performed immunohistochemistry in mouse livers. HMGB1 protein was found to localize predominantly in the nucleus of liver cells in the GFP-injected mice fed ND (Fig. 4c). However, HFD dramatically augmented the protein levels of HMGB1 in both nucleus and cytoplasm of liver cells in GFP-injected mice, which was markedly diminished by FBXW7 overexpression (Fig. 4c).

To further confirm the inhibitory effect of FBXW7 on the expression and release of hepatic HMGB1 under HFD conditions, shRNA-mediated knockdown of FBXW7 was performed in the mice fed with a HFD for10 weeks that exhibited a comparatively moderate metabolism disorders (Fig. 4d). Our results showed that Fbxw7 knockdown notably worsened hepatic inflammation and insulin resistance in HFD-fed mice (Additional file 3: Figure S3). Furthermore, Fbxw7 knockdown largely enhanced the protein expression of HMGB1, as well as its release from nucleus, in the livers of HFD-fed mice (Fig. 4e-f).

Collectively, these results indicate that FBXW7 ameliorates hepatic inflammation and consequent insulin resistance by suppressing the expression and release of HMGB1 in the HFD-fed mice.

### FBXW7 abates PKR signaling in HFD-fed mouse livers

**Fig. 5:**
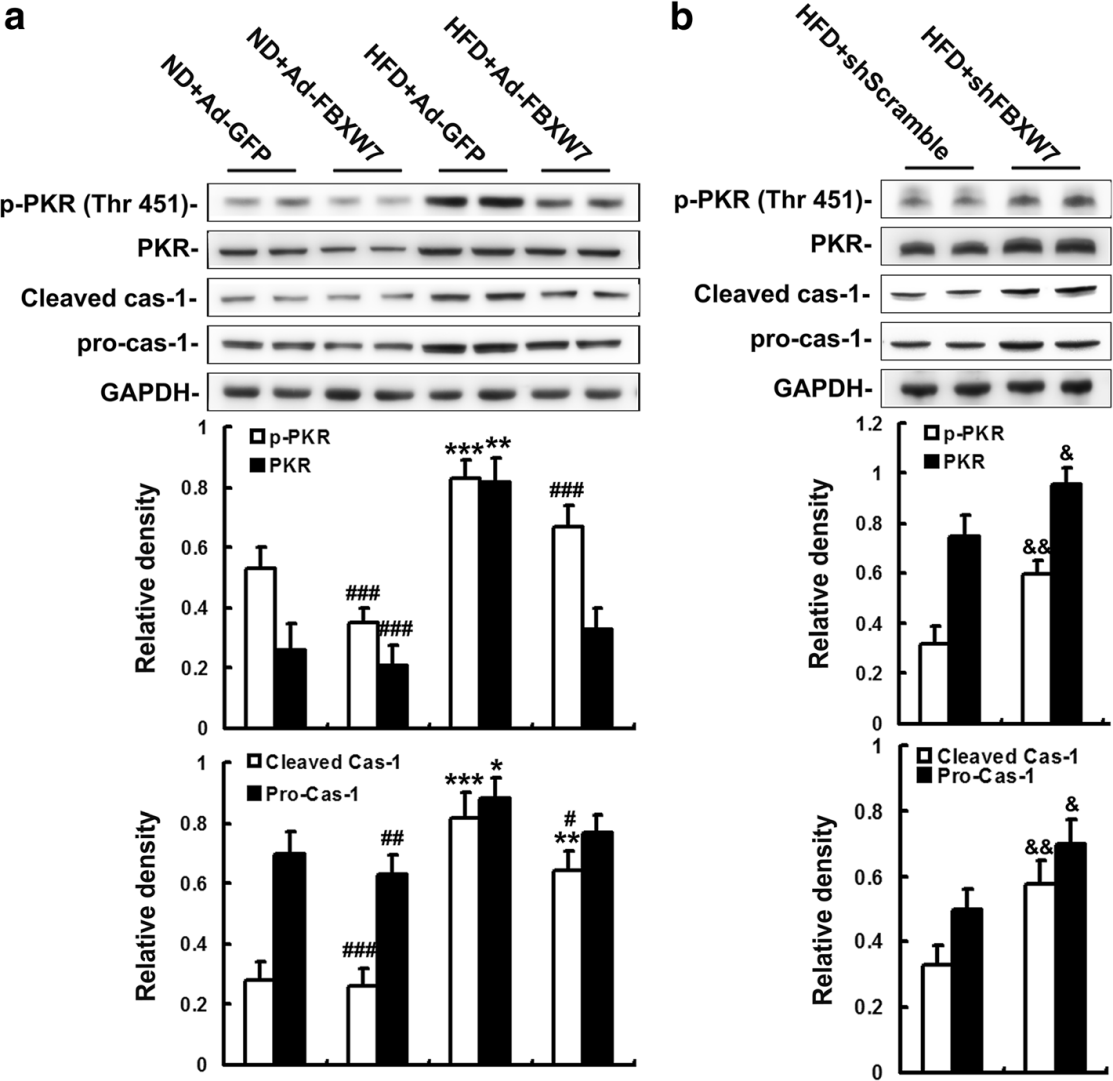
FBXW7 abates PKR signaling in HFD-fed mouse livers. **a** and (**b**) Immunoblotting and densitometry of the components of PKR signaling in mouse livers (*n* = 4). *P < 0.05, **P < 0.01, ***P < 0.001 versus the ND + Ad-GFP group; #*P* < 0.05, ##*P* < 0.01, ###*P* < 0.001 versus the HFD + Ad-GFP group; &*P* < 0.05, &&*P* < 0.01, versus the HFD + shScramble group

It is established that PKR provokes inflammasome-dependent HMGB1 release (Lu et al. 2012; Nakamura et al. 2010). Our results showed the activation of PKR, as well as its downstream target caspase-1, was largely enhanced in the livers of GFP-injected mice fed HFD, which was notably abated by FBXW7 overexpression (Fig. 5a). Interestingly, Fbxw7 knockdown remarkably augmented PKR activation and caspase-1 cleavage in the livers of HFD-fed mice (Fig. 5b).

These data suggest that FBXW7 inhibits PKR signaling to repress the expression and release of HMGB1 in the livers of HFD-fed mice.

### FBXW7 restrains TLR4 and RAGE signaling in HFD-fed mouse livers

**Fig. 6:**
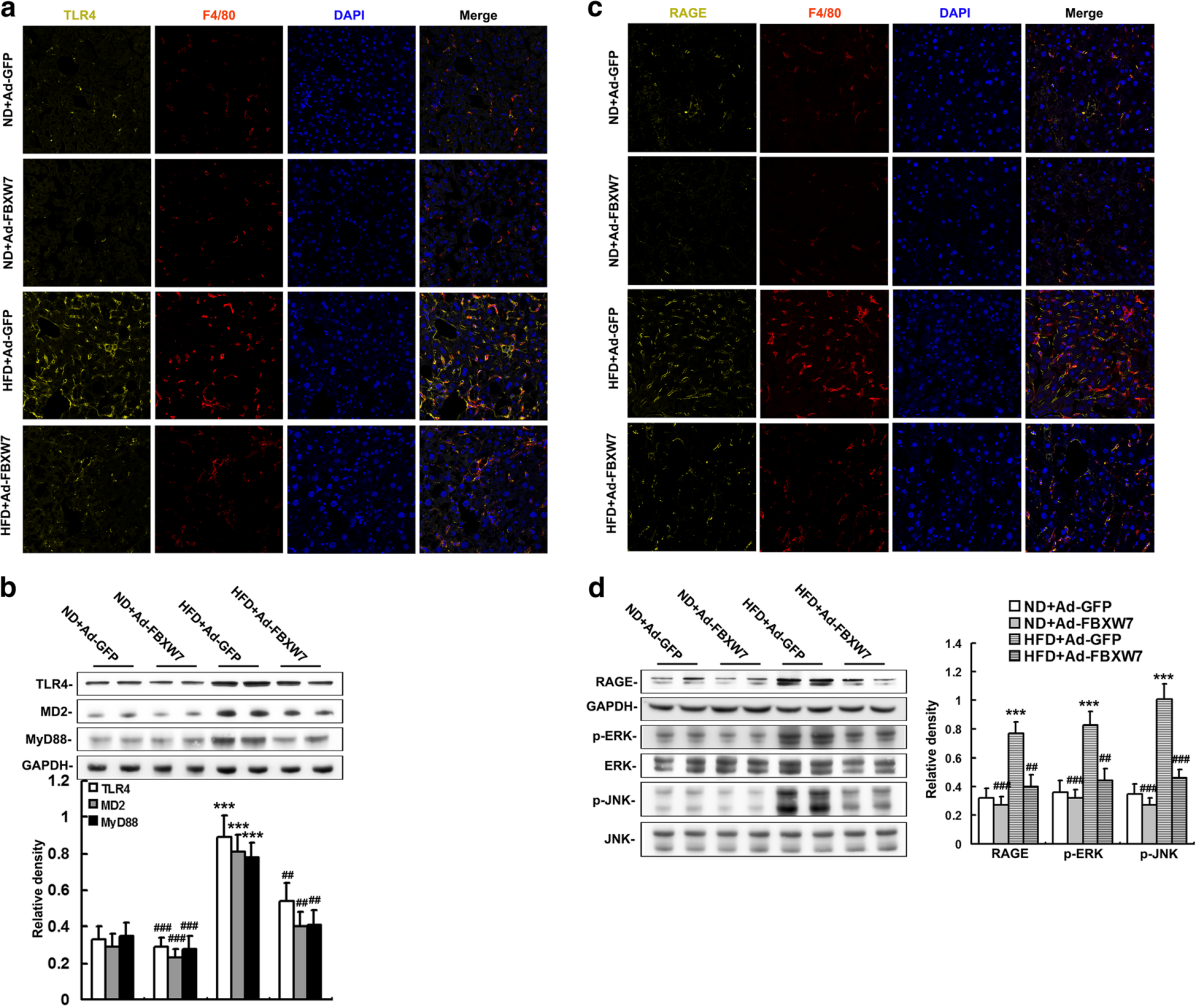
FBXW7 restrains TLR4 and RAGE signaling in HFD-fed mouse livers (*n* = 4). **a** Representative confocal immunofluorescence images of TLR4 (yellow), F4/80 (red) and DAPI (blue) in mouse livers. **b** Immunoblotting and densitometry of the components of TLR4 signaling in mouse livers. **c** Representative confocal immunofluorescence images of RAGE (yellow), F4/80 (red) and DAPI (blue) in mouse livers. **d** Immunoblotting and densitometry of the components of RAGE signaling in mouse livers. ***P < 0.001 versus the ND + Ad-GFP group; ##*P* < 0.01, ###*P* < 0.001 versus the HFD + Ad-GFP group

Substantial evidence reveals that TLR4 and RAGE, two essential components of innate immune, act as the dominant specific receptors for HMGB1 to trigger inflammatory response (Andersson et al. 2018). The results of western blot and immunofluorescence colocalization showed that the protein expression of TLR4 was significantly augmented in both Kupffer cells (F4/80 positive cells) and hepatocytes in the livers of GFP-injected mice fed a HFD compared to ND-fed controls (Fig. 6a, b). Interestingly, FBXW7 overexpression remarkably lowered TLR4 expression in the livers of HFD-fed mice (Fig. 6 a, b). MD2, a co-receptor for TLR4, facilitates HMGB1-mediated activation of TLR4 signaling (Yang et al. 2015). The results of western blot analysis revealed that the protein expression of MD2, as well as the major intracellular signaling cascades of TLR4 (MyD88), were notably increased in the livers of GFP-injected mice fed a HFD compared to ND-fed controls, which was effectively abated by FBXW7 overexpression (Fig. 6b). HFD also significantly elevated the protein expression of RAGE in both Kupffer cells and hepatocytes, as well as its major intracellular signaling cascades (p-ERK and p-JNK), in the livers of GFP-injected mice (Fig. 6c, d). However, FBXW7 overexpression dramatically reduced the protein expression of RAGE and its intracellular signaling cascades in the livers of HFD-fed mice (Fig. 6c, d).

In summary, these results indicate that FBXW7 restrains TLR4 and RAGE signaling in HFD-fed mouse livers.

### Exogenous HMGB1 abolishes FBXW7-mediated inhibition of TLR4 and RAGE signaling in HFD-fed mouse livers

**Fig. 7:**
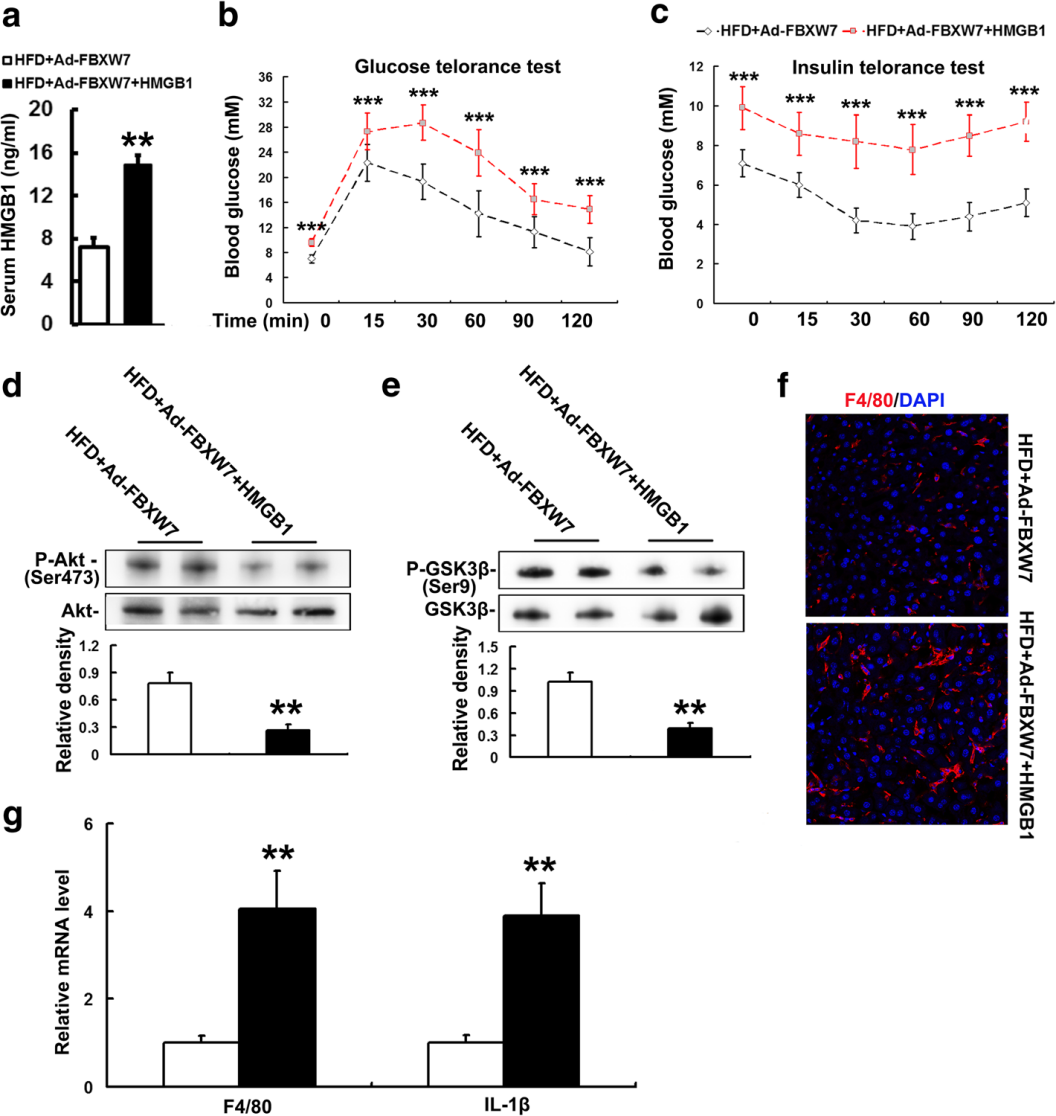
Exogenous HMGB1 abolishes FBXW7 overexpression-mediated improvement of insulin resistance and inflammation in HFD-fed mouse livers. **a** Serum HMGB1 levels in different treatment groups (*n* = 4). **b** Data of glucose tolerance tests in different treatment groups (*n* = 4). **c** Data of insulin tolerance tests in different treatment groups (*n* = 4). **d** Immunoblotting and densitometry of p-Akt in mouse livers (*n* = 4). **e** Immunoblotting and densitometry of p-GSK3β in mouse livers (*n* = 4). **f** Representative confocal immunofluorescence images of F4/80 (red) and DAPI (blue) in mouse livers (*n* = 4). **g** The mRNA level of inflammation-related genes in mouse livers (*n* = 3). All of the values are expressed as the mean ± SD. **P < 0.01, ***P < 0.001 versus the HFD + Ad-FBXW7 group

**Fig. 8:**
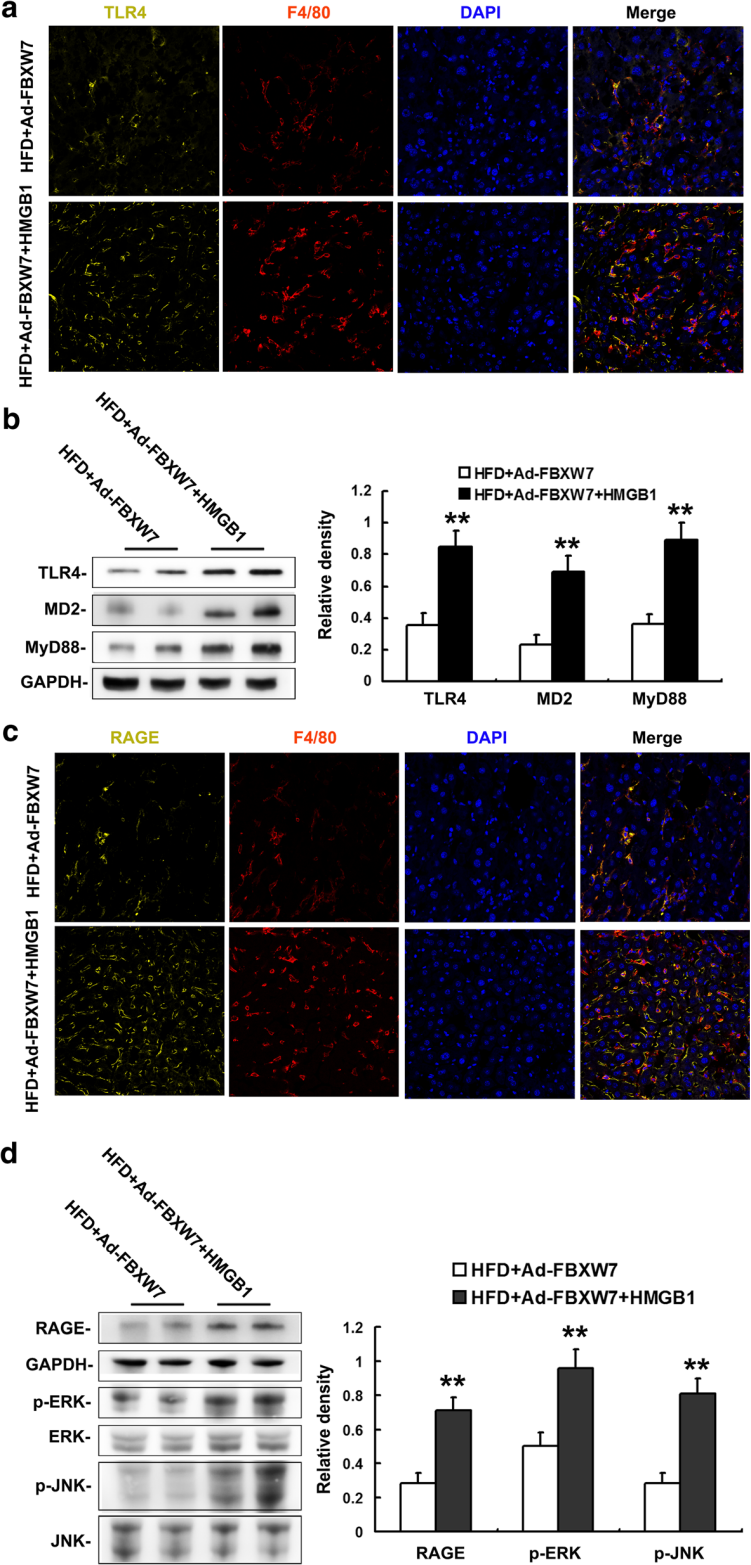
Exogenous HMGB1 abates FBXW7 overexpression-mediated inhibition of TLR4 and RAGE signaling in HFD-fed mouse livers (*n* = 4). **a** Representative confocal immunofluorescence images of TLR4 (yellow), F4/80 (red) and DAPI (blue) in mouse livers. **b** Immunoblotting and densitometry of the components of TLR4 signaling in mouse livers. **c** Representative confocal immunofluorescence images of RAGE (yellow), F4/80 (red) and DAPI (blue) in mouse livers. **d** Immunoblotting and densitometry of the components of RAGE signaling in mouse livers. All of the values are expressed as the mean ± SD. **P < 0.01 versus the versus the HFD + Ad-FBXW7 group

To further determine whether HMGB1 mediates the regulation of FBXW7 on TLR4 and RAGE signaling, recombinant HMGB1 or PBS were administered IP to FBXW7-injected mice fed a HFD. Recombinant HMGB1 effectively elevated the serum HMGB1 level in FBXW7-injected mice fed a HFD (Fig. 7a). Recombinant HMGB1 markedly enhanced inflammation as evident by an elevated expression of F4/80 and inflammation-related genes and insulin resistance characterized by an impairment of glucose tolerance and systemic insulin sensitivity and a decreased protein level of insulin signaling components in the livers of FBXW7-injected mice fed a HFD (Fig. 7b-g). Furthermore, recombinant HMGB1 significantly heightened the protein expression of TLR4, MD2 and MyD88 in the livers of FBXW7-injected mice fed a HFD (Fig. 8a, b). Meanwhile, recombinant HMGB1 notably augmented the protein expression of RAGE and its major intracellular signaling cascades (ERK and JNK) in the livers of FBXW7-injected mice fed a HFD (Fig. 8c, d).

In combination with the above mentioned results, these results suggest that FBXW7 represses TLR4 and RAGE signaling by inhibiting the expression and release of HMGB1 in HFD-fed mouse livers.

## Discussion

Improving hepatic insulin resistance is a therapeutic strategy for NAFLD, especially for ‘metabolic’ NAFLD that develops because of excessive caloric intake rather than genetic risks (Cipriani et al. 2010; Gastaldelli 2017). Among a variety of contributing factors, metaflammation plays an important role in the development of obesity-related hepatic insulin resistance (Perry et al. 2015; Saberi et al. 2009). It is established that innate immune activation plays a key role in provoking inflammatory response under NAFLD (Cai et al. 2018; Luo et al. 2018; Nati et al. 2016). However, the pathogenesis of innate immune activation under NAFLD conditions is still incompletely understood. In the present study, we identified FBXW7 as an important mediator in the pathogenesis of NAFLD by controlling HMGB1-mediated innate immune signaling. We revealed that FBXW7 effectively abated the expression and release of HMGB1, thereby suppressing TLR4 and RAGE signaling to attenuate inflammation and consequent insulin resistance, ultimately ameliorating NAFLD.

In addition to controlling metabolism reprogramming in cancer, FBXW7 is implicated in the maintenance of glucose and lipid homeostasis during metabolic syndrome, such as mediating lipid metabolism by triggering SREBP1 degradation, facilitating glucose homeostasis through promoting fetuin A degradation and perturbing systemic lipid and glucose metabolism via targeting REV-ERBα for degradation, which may contribute to the development of NAFLD (Onoyama et al. 2011; Tu et al. 2012; Zhao et al. 2016, 2018). Moreover, liver-specific Fbxw7 knockout causes enhancement of hepatic lipogenesis and steatosis in mice (Onoyama et al. 2011; Zhao et al. 2016, 2018). These studies indicate that Fbxw7 is a potential regulator of NAFLD development. This study showed that FBXW7 overexpression improved HFD-induced NAFLD and related metabolic parameters in mice, confirming its regulatory role in NAFLD development. During NAFLD, the excessive hepatic fat accumulation leads to liver cells stress, damage and death, which releases danger signals, thereby activating innate immune signaling to trigger inflammatory response (Csak et al. 2011; Ganz et al. 2015). HFD can also alter the composition of gut microbiota and disrupt intestinal barrier integrity, which elevates the serum levels of bacterial products (eg, LPS), triggering innate immune activation-mediated hepatic inflammation (Henao-Mejia et al. 2012; Porras et al. 2017). The inflammatory milieu heightens the expression and release of proinflammatory cytokines, such as TNFα and IL-1β, which play a key role in damnification of insulin signaling, leading to insulin resistance and subsequently promoting NAFLD. Our results revealed that FBXW7 overexpression attenuated hepatic inflammatory response and insulin resistance in HFD-fed mice, while FBXW7 knockdown exacerbated these disorders. Thus, our results reveals that the protective effects of FBXW7 on NAFLD is associated with suppression of hepatic inflammation and consequent insulin resistance.

Recently, growing evidence highlights that HMGB1 plays a crucial role in triggering innate immune activation-associated inflammation including metaflammation through being released into extracellular milieu to act as a key DAMP (Li et al. 2011; Lotze and Tracey 2005; Lundbäck et al. 2016). It is well established that HMGB1 is an important innate alarmin responsible for the activation of hepatic macrophage to promote the expression and secretion of proinflammatory cytokines, which play a pivotal role in the initiation of metaflammation during NAFLD (Zhang et al. 2013a). Furthermore, a number of studies demonstrate that HMGB1 is released by liver parenchymal cells, leading to TLR4/MyD88-mediated cytokines release from hepatocytes in the early stage of NAFLD, which precedes the occurrence of kupffer cell-mediated inflammation (Li et al. 2011). These studies indicate that HMGB1 may contribute to the early development of NAFLD by triggering the release of cytokines from hepatocytes, which disturbs hepatic metabolism and immune response, leading to the development and progression of NAFLD. In the present study, our results showed that FBXW7 overexpression dramatically attenuated, while FBXW7 knockdown augmented, the expression and release of HMGB1 in the livers of HFD-fed mice. Moreover, Exogenous HMGB1 treatment abolished FBXW7-mediated inhibition of hepatic inflammation and insulin resistance in HFD-fed mouse livers. Thus, this study provided experimental evidence that FBXW7 ameliorated hepatic inflammation and insulin resistance by suppressing the expression and release of HMGB1 in NAFLD. Upon obesity or HFD feeding, many exogenous and endogenous stimuli, such as bacterial endotoxin, cytokines, cell stress and damage, provoke the expression and secretion of HMGB1. Though the mechanism underlying HMGB1 release during metaflammation is still obscure, some studies demonstrate that PKR, activated in liver tissues upon obesity and HFD feeding, triggers inflammasome-dependent HMGB1 release (Lu et al. 2012; Nakamura et al. 2010). In the present study, HFD markedly elevated PKR activation and consequent caspase-1 cleavage, which was significantly suppressed by FBXW7 expression, but enhanced by FBXW7 knockdown in mouse livers. Our results suggest that PKR inhibition may be involved in the repressive effects of FBXW7 on HMGB1 release in NAFLD. PKR is recognized as an important linkage integrating nutrient, stress and metabolic disorder and is well demonstrated to be activated by ER stress (Chang et al. 2017; Lu et al. 2012). It is widely reported that mTOR, an important target of FBXW7, provokes ER stress in obese mice (Li et al. 2014). Thus, FBXW7-mediated mTOR degradation may be a possible pathway for the inhibition of PKR activation and HMGB1 release in the livers of HFD-fed mice. This interesting possibility needs further investigation.

After secretion into extracellular milieu, HMGB1 binds to a variety of cell surface receptors, such as TLRs, cluster of differentiation 24 (CD24)/Siglec-10 and RAGE, to activate innate immune signaling regulating inflammatory response in both immune and somatic cells (Andersson et al. 2018; Fiuza et al. 2003; Lotze and Tracey 2005). It is well established that TLR4 and RAGE are two dominant HMGB1-receptors triggering inflammation during various inflammatory diseases (Fiuza et al. 2003; Lotze and Tracey 2005; Nogueira-Machado et al. 2011). It is also indicated that HMGB1/TLR4 and HMGB1/RAGE signaling may play a role in the pathogenesis of NAFLD (Li et al. 2011; Jeon et al. 2014). TLR4 is required for HMGB1-induced cytokine production, which is facilitated by its co-receptor MD2 (He et al. 2018; Yang et al. 2015). HMGB1-RAGE axis has been extensively studied to induce proinflammatory cytokine production, which is mainly mediated by MAPK signaling pathway (Jhun et al. 2015). Contrarily, inhibition of TLR4 and/or RAGE effectively attenuates HMGB1-mediated inflammation (Fiuza et al. 2003; Nogueira-Machado et al. 2011). These studies verify a key role of TLR4 and/or RAGE in the HMGB1-mediated inflammation. In this study, FBXW7 overexpression dramatically reduced the protein expression of TLR4, RAGE and their intracellular signaling cascades in the livers of HFD-fed mice. Moreover, exogenous HMGB1 treatment abrogated FBXW7-mediated inhibition of TLR4 and RAGE signaling in HFD-fed mouse livers. Thus, our results reveal that FBXW7 restrains TLR4 and RAGE signaling at least in part by inhibiting the expression and release of HMGB1 in HFD-fed mouse livers.

## Conclusions

In conclusion, this study demonstrates a protective role of FBXW7 in the development and progression of NAFLD by attenuating inflammation and consequent insulin resistance, which is associated with the suppression of HMGB1-mediated innate immune signaling. Our study provides the experimental evidence suggesting that FBXW7 is a potential target for therapeutic intervention in NAFLD development.

## Additional files

Additional file 1: **Figure S1.** The protein levels of FBXW7 are not significantly increased in other metabolic tissues of Ad-FBXW7-injected mice. (a) Immunoblotting and densitometry of FBXW7 and GFP in mouse adipose tissues (*n* = 4). (b) Immunoblotting and densitometry of FBXW7 and GFP in mouse muscles (n = 4). All of the values are expressed as the mean ± SD. *P < 0.05, **P < 0.01, ***P < 0.001 versus the ND + Ad-GFP group; #*P* < 0.05, ###*P* < 0.001 versus the HFD + Ad-GFP group. (TIF 2441 kb)

Additional file 2: **Figure S2.** FBXW7 overexpression don’t notably affect body weight and food intake in mice. (a) Body weight in mice (*n* = 8). (b) Food intake in mice (n = 8). All of the values are expressed as the mean ± SD. ***P < 0.001 versus the ND + Ad-GFP group. (TIF 1378 kb)

Additional file 3: **Figure S3.** FBXW7 knockdown worsens insulin resistance and inflammation in HFD-fed mouse livers. (a) Data of glucose tolerance tests in different treatment groups (*n* = 4). (b) Data of insulin tolerance tests in different treatment groups (*n* = 4). (c) Immunoblotting and densitometry of p-Akt in mouse livers (*n* = 4). (d) Immunoblotting and densitometry of p-GSK3β in mouse livers (*n* = 4). (e) Representative confocal immunofluorescence images of F4/80 (red) and DAPI (blue) in mouse livers (*n* = 4). (f) The mRNA level of inflammation-related genes in mouse livers (*n* = 3). All of the values are expressed as the mean ± SD. *P < 0.05, **P < 0.01, ***P < 0.001 versus the HFD + shScramble group. (TIF 3101 kb)

## Data Availability

All data supporting the conclusions of this study are included within the article and its additional files.

## Abbreviations

ALT: Alanine aminotransferase
C/EBPδ: CCAAT/enhancer binding protein delta
DAMPs: Damage-associated molecular patterns
ERK: Extracellular-signal-regulated kinase
FBXW7: F-box- and WD repeat domain-containing 7
GAPDH: Glyceraldehyde-3-phosphate dehydrogenase
GFP: Green fluorescent protein
GSK-3β: Glycogen synthase kinase-3
HFD: High-fat diet
HMGB1: High-mobility group box 1 protein
i.p.: Intraperitoneal
IL-1β: Interleukin-1β
ITT: Insulin tolerance tests
MCP-1: Monocyte chemoattractant protein-1
MD2: Myeloid differentiation factor 2
Mtor: Mammalian target of rapamycin
MyD88: Myeloid differentiation factor 88
NAFLD: Nonalcoholic fatty liver disease
ND: Normal diet
NF-κB: Nuclear factor kappa B
OGTT: Oral glucose tolerance tests
PFU: Plaque forming units
PKR: Protein kinase R
RAGE: Receptor for advanced glycation end products
TG: Triglyceride
TLR4: Toll-like receptor 4
TNF-α: Tumor necrosis factor α

## References

[CR1] Afrin R, Arumugam S, Rahman A, Wahed MI, Karuppagounder V, Harima M (2017). Curcumin ameliorates liver damage and progression of NASH in NASH-HCC mouse model possibly by modulating HMGB1-NF-κB translocation. Int Immunopharmacol.

[CR2] Andersson U, Yang H, Harris H (2018). High-mobility group box 1 protein (HMGB1) operates as an alarmin outside as well as inside cells. Semin Immunol.

[CR3] Balamurugan K, Sharan S, Klarmann KD, Zhang Y, Coppola V, Summers GH (2013). FBXW7α attenuates inflammatory signalling by downregulating C/EBPδ and its target gene Tlr4. Nat Commun.

[CR4] Cai J, Zhang XJ, Li H (2018). Role of innate immune signaling in non-alcoholic fatty liver disease. Trends Endocrinol Metab.

[CR5] Chang SH, Huang SW, Wang ST, Chung KC, Hsieh CW, Kao JK (2017). Imiquimod-induced autophagy is regulated by ER stress-mediated PKR activation in cancer cells. J Dermatol Sci.

[CR6] Cipriani S, Mencarelli A, Palladino G, Fiorucci S (2010). FXR activation reverses insulin resistance and lipid abnormalities and protects against liver steatosis in Zucker (fa/fa) obese rats. J Lipid Res.

[CR7] Csak T, Ganz M, Pespisa J, Kodys K, Dolganiuc A, Szabo G (2011). Fatty acid and endotoxin activate inflammasomes in mouse hepatocytes that release danger signals to stimulate immune cells. Hepatology..

[CR8] De Lorenzo G, Ferrari S, Cervone F, Okun E (2018). Extracellular DAMPs in plants and mammals: immunity, tissue damage and repair. Trends Immunol.

[CR9] Entezari M, Javdan M, Antoine DJ, Morrow DM, Sitapara RA, Patel V (2014). Inhibition of extracellular HMGB1 attenuates hyperoxia-induced inflammatory acute lung injury. Redox Biol.

[CR10] Fiuza C, Bustin M, Talwar S, Tropea M, Gerstenberger E, Shelhamer JH (2003). Inflammation-promoting activity of HMGB1 on human microvascular endothelial cells. Blood..

[CR11] Folch J, Lees M, Sloane Stanley GH (1957). A simple method for the isolation and purification of total lipides from animal tissues. J Biol Chem.

[CR12] Ganz M, Bukong TN, Csak T, Saha B, Park JK, Ambade A (2015). Progression of non-alcoholic steatosis to steatohepatitis and fibrosis parallels cumulative accumulation of danger signals that promote inflammation and liver tumors in a high fat-cholesterol-sugar diet model in mice. J Transl Med.

[CR13] Gastaldelli A (2017). Insulin resistance and reduced metabolic flexibility: cause or consequence of NAFLD?. Clin Sci (Lond).

[CR14] He M, Bianchi ME, Coleman TR, Tracey KJ, Al-Abed Y (2018). Exploring the biological functional mechanism of the HMGB1/TLR4/MD-2 complex by surface plasmon resonance. Mol Med.

[CR15] Henao-Mejia J, Elinav E, Jin C, Hao L, Mehal WZ, Strowig T (2012). Inflammasome-mediated dysbiosis regulates progression of NAFLD and obesity. Nature..

[CR16] Jelenik T, Kaul K, Séquaris G, Flögel U, Phielix E, Kotzka J (2017). Mechanisms of insulin resistance in primary and secondary nonalcoholic fatty liver. Diabetes..

[CR17] Jeon BT, Heo RW, Shin HJ, Yi CO, Lee YH, Joung HN (2014). Attenuation by a Vigna nakashimae extract of nonalcoholic fatty liver disease in high-fat diet-fed mice. Biosci Biotechnol Biochem.

[CR18] Jhun J, Lee S, Kim H, Her YM, Byun JK, Kim EK (2015). HMGB1/RAGE induces IL-17 expression to exaggerate inflammation in peripheral blood cells of hepatitis B patients. J Transl Med.

[CR19] Kanatsu-Shinohara M, Onoyama I, Nakayama KI, Shinohara T (2014). Skp1-Cullin-F-box (SCF)-type ubiquitin ligase FBXW7 negatively regulates spermatogonial stem cell self-renewal. Proc Natl Acad Sci U S A.

[CR20] Ke B, Zhao Z, Ye X, Gao Z, Manganiello V, Wu B (2015). Inactivation of NF-κB p65 (RelA) in liver improves insulin sensitivity and inhibits cAMP/PKA pathway. Diabetes..

[CR21] Kourtis N, Moubarak RS, Aranda-Orgilles B, Lui K, Aydin IT, Trimarchi T (2015). FBXW7 modulates cellular stress response and metastatic potential through HSF1 post-translational modification. Nat Cell Biol.

[CR22] Li H, Min Q, Ouyang C, Lee J, He C, Zou MH (2014). AMPK activation prevents excess nutrient-induced hepatic lipid accumulation by inhibiting mTORC1 signaling and endoplasmic reticulum stress response. Biochim Biophys Acta.

[CR23] Li L, Chen L, Hu L, Liu Y, Sun HY, Tang J (2011). Nuclear factor high-mobility group box 1 mediating the activation of toll-like receptor 4 signaling in hepatocytes in the early stage of nonalcoholic fatty liver disease in mice. Hepatology..

[CR24] Lotze MT, Tracey KJ (2005). High-mobility group box 1 protein (HMGB1): nuclear weapon in the immune arsenal. Nat Rev Immunol.

[CR25] Lu B, Antoine DJ, Kwan K, Lundbäck P, Wähämaa H, Schierbeck H (2014). JAK/STAT1 signaling promotes HMGB1 hyperacetylation and nuclear translocation. Proc Natl Acad Sci U S A.

[CR26] Lu B, Nakamura T, Inouye K, Li J, Tang Y, Lundbäck P (2012). Novel role of PKR in inflammasome activation and HMGB1 release. Nature..

[CR27] Lundbäck P, Lea JD, Sowinska A, Ottosson L, Fürst CM, Steen J, Aulin C, Clarke JI, Kipar A, Klevenvall L, Yang H, Palmblad K, Park BK, Tracey KJ, Blom AM, Andersson U, Antoine DJ, Harris HE (2016). A novel high mobility group box 1 neutralizing chimeric antibody attenuates drug-induced liver injury and postinjury inflammation in mice. Hepatology.

[CR28] Luo X, Li H, Ma L, Zhou J, Guo X, Woo SL (2018). Expression of STING is increased in liver tissues from patients with NAFLD and promotes macrophage-mediated hepatic inflammation and fibrosis in mice. Gastroenterology..

[CR29] Mihm S (2018). Danger-associated molecular patterns (DAMPs): molecular triggers for sterile inflammation in the liver. Int J Mol Sci.

[CR30] Nakamura T, Furuhashi M, Li P, Cao H, Tuncman G, Sonenberg N (2010). Double-stranded RNA-dependent protein kinase links pathogen sensing with stress and metabolic homeostasis. Cell..

[CR31] Nati M, Haddad D, Birkenfeld AL, Koch CA, Chavakis T, Chatzigeorgiou A (2016). The role of immune cells in metabolism-related liver inflammation and development of non-alcoholic steatohepatitis (NASH). Rev Endocr Metab Disord.

[CR32] Nogueira-Machado JA, Volpe CM, Veloso CA, Chaves MM (2011). HMGB1, TLR and RAGE: a functional tripod that leads to diabetic inflammation. Expert Opin Ther Targets.

[CR33] Onoyama I, Suzuki A, Matsumoto A, Tomita K, Katagiri H, Oike Y (2011). Fbxw7 regulates lipid metabolism and cell fate decisions in the mouse liver. J Clin Invest.

[CR34] Perry RJ, Camporez JG, Kursawe R, Titchenell PM, Zhang D, Perry CJ (2015). Hepatic acetyl CoA links adipose tissue inflammation to hepatic insulin resistance and type 2 diabetes. Cell..

[CR35] Porras D, Nistal E, Martínez-Flórez S, Pisonero-Vaquero S, Olcoz JL, Jover R (2017). Protective effect of quercetin on high-fat diet-induced non-alcoholic fatty liver disease in mice is mediated by modulating intestinal microbiota imbalance and related gut-liver axis activation. Free Radic Biol Med.

[CR36] Saberi M, Woods NB, de Luca C, Schenk S, Lu JC, Bandyopadhyay G (2009). Hematopoietic cell-specific deletion of toll-like receptor 4 ameliorates hepatic and adipose tissue insulin resistance in high-fat-fed mice. Cell Metab.

[CR37] Tilg H, Hotamisligil GS (2006). Nonalcoholic fatty liver disease: cytokine-adipokine interplay and regulation of insulin resistance. Gastroenterology..

[CR38] Tu K, Zheng X, Yin G, Zan X, Yao Y, Liu Q (2012). Evaluation of Fbxw7 expression and its correlation with expression of SREBP-1 in a mouse model of NAFLD. Mol Med Rep.

[CR39] Yang H, Wang H, Ju Z, Ragab AA, Lundbäck P, Long W (2015). MD-2 is required for disulfide HMGB1-dependent TLR4 signaling. J Exp Med.

[CR40] Zeng W, Shan W, Gao L, Gao D, Hu Y, Wang G (2015). Inhibition of HMGB1 release via salvianolic acid B-mediated SIRT1 up-regulation protects rats against non-alcoholic fatty liver disease. Sci Rep.

[CR41] Zhang W, Wang LW, Wang LK, Li X, Zhang H, Luo LP (2013). Betaine protects against high-fat-diet-induced liver injury by inhibition of high-mobility group box 1 and toll-like receptor 4 expression in rats. Dig Dis Sci.

[CR42] Zhang ZF, Fan SH, Zheng YL, Lu J, Wu DM, Shan Q (2014). Troxerutin improves hepatic lipid homeostasis by restoring NAD(+)-depletion-mediated dysfunction of lipin 1 signaling in high-fat diet-treated mice. Biochem Pharmacol.

[CR43] Zhang ZF, Lu J, Zheng YL, Wu DM, Hu B, Shan Q (2013). Purple sweet potato color attenuates hepatic insulin resistance via blocking oxidative stress and endoplasmic reticulum stress in high-fat-diet-treated mice. J Nutr Biochem.

[CR44] Zhao J, Xiong X, Li Y, Liu X, Wang T, Zhang H (2018). Hepatic F-box protein FBXW7 maintains glucose homeostasis through degradation of fetuin-A. Diabetes..

[CR45] Zhao X, Hirota T, Han X, Cho H, Chong LW, Lamia K (2016). Circadian amplitude regulation via FBXW7-targeted REV-ERBα degradation. Cell..

