# Supplementary figures and images for: FBXW7 suppresses HMGB1-mediated innate immune signaling to attenuate hepatic inflammation and insulin resistance in a mouse model of nonalcoholic fatty liver disease
Source: Mol Med. 2019 Jun 18;25:29. doi: 10.1186/s10020-019-0099-9 (PMC6582600; doi:10.1186/s10020-019-0099-9)

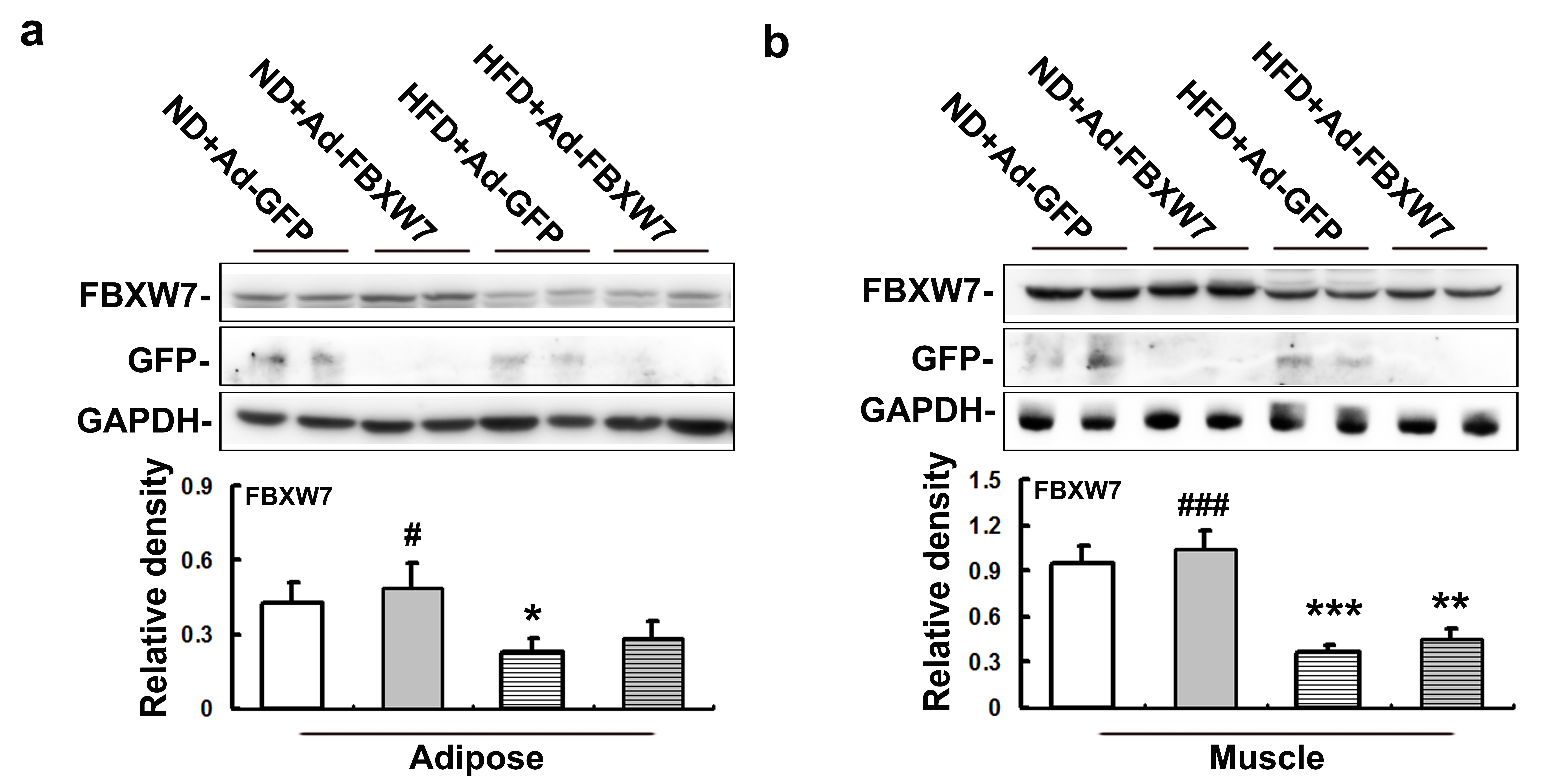

Supplement: Supplementary file 1 — Figure S1. The protein levels of FBXW7 are not significantly increased in other metabolic tissues of Ad-FBXW7-injected mice. (a) Immunoblotting and densitometry of FBXW7 and GFP in mouse adipose tissues (n = 4). (b) Immunoblotting and densitometry of FBXW7 and GFP in mouse muscles (n = 4). All of the values are expressed as the mean ± SD. *P < 0.05, **P < 0.01, ***P < 0.001 versus the ND + Ad-GFP group; #P < 0.05, ###P < 0.001 versus the HFD + Ad-GFP group. (TIF 2441 kb) [file 10020_2019_99_MOESM1_ESM.tif]

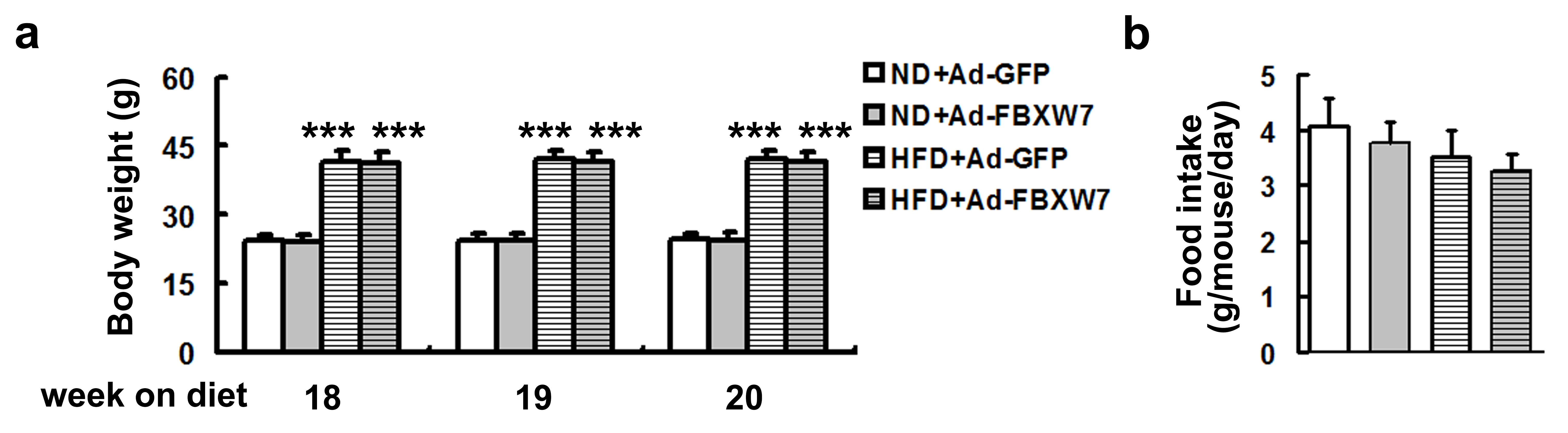

Supplement: Supplementary file 2 — Figure S2. FBXW7 overexpression don’t notably affect body weight and food intake in mice. (a) Body weight in mice (n = 8). (b) Food intake in mice (n = 8). All of the values are expressed as the mean ± SD. ***P < 0.001 versus the ND + Ad-GFP group. (TIF 1378 kb) [file 10020_2019_99_MOESM2_ESM.tif]

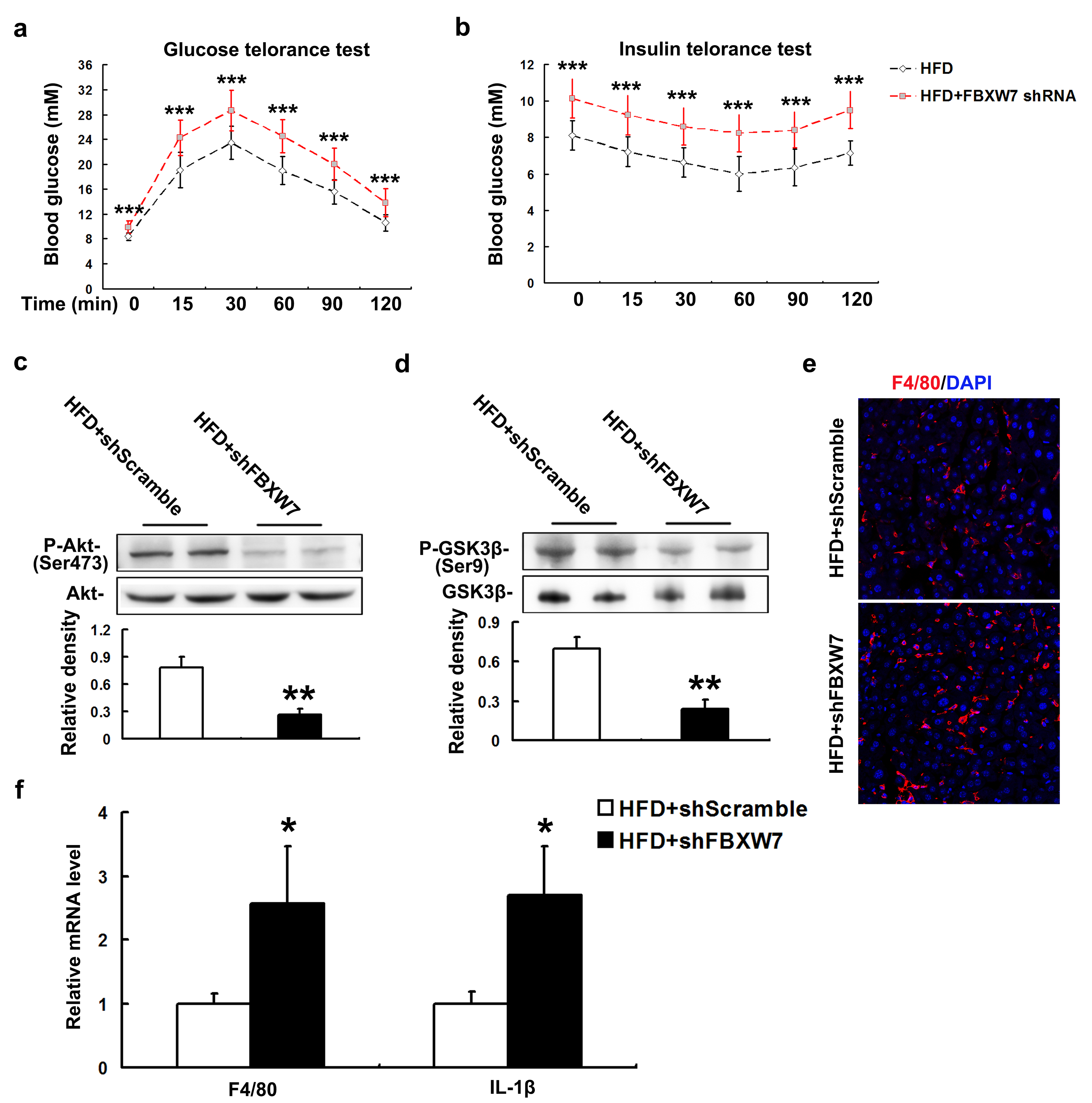

Supplement: Supplementary file 3 — Figure S3. FBXW7 knockdown worsens insulin resistance and inflammation in HFD-fed mouse livers. (a) Data of glucose tolerance tests in different treatment groups (n = 4). (b) Data of insulin tolerance tests in different treatment groups (n = 4). (c) Immunoblotting and densitometry of p-Akt in mouse livers (n = 4). (d) Immunoblotting and densitometry of p-GSK3β in mouse livers (n = 4). (e) Representative confocal immunofluorescence images of F4/80 (red) and DAPI (blue) in mouse livers (n = 4). (f) The mRNA level of inflammation-related genes in mouse livers (n = 3). All of the values are expressed as the mean ± SD. *P < 0.05, **P < 0.01, ***P < 0.001 versus the HFD + shScramble group. (TIF 3101 kb) [file 10020_2019_99_MOESM3_ESM.tif]
